# A mechanistic stochastic framework for regulating bacterial cell division

**DOI:** 10.1038/srep30229

**Published:** 2016-07-26

**Authors:** Khem Raj Ghusinga, Cesar A. Vargas-Garcia, Abhyudai Singh

**Affiliations:** 1 Department of Electrical and Computer Engineering, University of Delaware, Newark, DE 19716, USA; 2 Department of Biomedical Engineering, University of Delaware, Newark, DE 19716, USA; 3 Department of Mathematical Sciences, University of Delaware, Newark, DE 19716, USA

## Abstract

How exponentially growing cells maintain size homeostasis is an important fundamental problem. Recent single-cell studies in prokaryotes have uncovered the adder principle, where cells add a fixed size (volume) from birth to division, irrespective of their size at birth. To mechanistically explain the adder principle, we consider a timekeeper protein that begins to get stochastically expressed after cell birth at a rate proportional to the volume. Cell-division time is formulated as the first-passage time for protein copy numbers to hit a fixed threshold. Consistent with data, the model predicts that the noise in division timing increases with size at birth. Intriguingly, our results show that the distribution of the volume added between successive cell-division events is independent of the newborn cell size. This was dramatically seen in experimental studies, where histograms of the added volume corresponding to different newborn sizes collapsed on top of each other. The model provides further insights consistent with experimental observations: the distribution of the added volume when scaled by its mean becomes invariant of the growth rate. In summary, our simple yet elegant model explains key experimental findings and suggests a mechanism for regulating both the mean and fluctuations in cell-division timing for controlling size.

Recurring cycles of growth and division of a cell is a ubiquitous theme across all organisms. How an isogenic population of exponentially growing cells maintains a narrow distribution of cell size, a property known as size homeostasis, has been extensively studied, e.g., see^1^,^2^,^3^,^4^ and references therein. From a phenomenological standpoint, recent experiments reveal that diverse microorganisms achieve size homeostasis via an adder principle^5^,^6^,^7^,^8^. As per this strategy, cells add a constant size from birth to division regardless of their size at birth^9^,^10^. Interestingly, the size accumulated by a single cell between birth and division exhibits considerable cell-to-cell differences, and these differences follow unique statistical properties. For example, in a given growth condition, the added size is drawn from a fixed probability distribution independent of the newborn cell size. Moreover, the distribution of the added size normalized by its mean is invariant across growth conditions^6^. Here, we explore biophysical models that lead to the adder principle of cell size control and provide insights into its statistical properties.

To realize the adder principle mechanistically, a cell needs to somehow track the size it has accumulated since the previous division and trigger the next division upon addition of the desired size. One biophysical model proposed to achieve this assumes a protein which begins to get expressed right after cell birth at a rate proportional to instantaneous volume (size). The cell grows exponentially over time and division is triggered when protein copy numbers reach a critical threshold after which the protein is assumed to degrade (Fig. 1a)^7^,^10^,^11^. Such copy number dependent triggering of cell division could potentially be implemented via the localization of protein into compartments whose volume does not change appreciably with the cell volume^12^. Moreover, the synthesis and the degradation of the protein in this model are used in broad sense; they could as well be activation of timekeeper proteins in size dependent manner, and deactivation after triggering of division. While this deterministic model results in a constant size added from cell birth to division^10^,^11^, it remains to be seen how noise mechanisms can be incorporated in this model to explain statistical fluctuations in cell size. A plausible source of noise could be the inherent stochastic nature of protein expression that has been universally observed in prokaryotes and eukaryotes^13^,^14^,^15^,^16^,^17^. Such stochasticity in protein synthesis is amplified at the level of individual cells, where gene products are often present at low molecular counts.

Considering noisy expression of the timekeeper protein, one can formulate cell-division time as a first-passage time problem: an event (division) occurs when a stochastic process (protein copy numbers) hits a threshold for the first time (Fig. 1b). Exploiting this first-passage time framework, we derive an exact analytical formula for the cell-division time distribution for a given newborn cell size. Consistent with data, these results predict that the mean cell-division time decreases with increasing cell size at birth, and the randomness (quantified by coefficient of variation squared) in the cell-division time increases with newborn cell size. Intriguingly, analysis of the model further shows that the distribution of the volume added from cell birth to division is always independent of the newborn cell size. Finally, we find that the distributions of added volume and cell division time have scale invariant forms: distributions in different growth conditions collapse upon each other after rescaling them with their respective means. We discuss potential candidates for the timekeeper protein and deliberate upon model modifications that result in deviations from the adder principle.

## Results

### Model description

Consider a newborn cell with volume *V*_*b*_ at time *t* = 0. Its volume at a time *t* after birth is given by *V*(*t*) = *V*_*b*_ exp(*αt*), where *α* > 0 represents the growth rate. After cell birth, the timekeeper protein begins to get transcribed at a rate *r*(*t*) = *k*_*m*_*V*(*t*), where *k*_*m*_ is the transcription rate in the concentration sense. Note that this scaling of protein synthesis with instantaneous cell volume is essential for preserving gene product concentrations in growing cells. In the stochastic formulation, the probability of a transcription event occurring in an infinitesimal time interval (*t*, *t* + *dt*] is given by *r*(*t*)*dt*. Assuming short-lived mRNAs, each transcript degrades instantaneously after producing a burst of protein molecules^18^,^19^,^20^,^21^,^22^,^23^. Stochastic expression of the timekeeper protein is compactly represented by the following biochemical reaction:


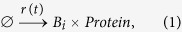


where *r*(*t*) = *k*_*m*_*V*(*t*) can be interpreted as the burst arrival rate and *B*_*i*_, *i* ∈ {1, 2, 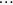}, are identical and independent random variables denoting the size of protein bursts with mean *b* := 〈*B*_*i*_〉. The burst size represents the number of protein molecules synthesized in a single mRNA lifetime and typically follows a geometric distribution^19^,^21^,^23^,^24^,^25^,^26^. However, to allow a wide range of protein accumulation processes to be covered by equation (1), we assume that *B*_*i*_ follows an arbitrary non-negative integer-valued distribution. One example of such a mechanism could be to consider a protein *A* whose concentration is constant throughout the cell cycle. This protein is stochastically converted to an active form A^*^ at a rate proportional to the number of molecules of *A*. In essence, this can be thought of as production of A^*^ in bursts which takes place at a rate proportional to the cell volume.

Let *x*(*t*) denote the number of timekeeper molecules in the cell at time *t* after birth. Assuming a stable protein with no active proteolysis, we have 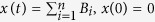, where *n* is the number of bursts (transcription events) in [0, *t*]. Cell division occurs when *x*(*t*) reaches a threshold *X* and the protein is degraded (or deactivated) thereafter. Given this timing mechanism, cell-division time can be mathematically represented as the first-passage time (*FPT*).


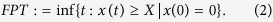


This first-passage time framework assumes that cell division occurs upon precise attainment of *X* protein molecules. In principle, one could generalize equation (2) by defining a monotonically increasing function *h*(*x*) that defines a probabilistic rate of cell division at time *t* given *x*(*t*) molecules. Interestingly, analysis reveals that the average size added from birth to division is invariant of the newborn cell size *V*_*b*_ iff


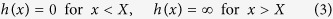


(see Supplementary Information (SI), section S1). Thus, a sharp threshold, where cell division cannot be triggered before attainment of a precise number of molecules seems to be a necessary ingredient of the adder principle.

### Distribution of the cell-division time given newborn cell size

Here we derive the distribution of the cell-division time (*FPT*) for a given newborn cell size *V*_*b*_ and investigate how its statistical moments depend on *V*_*b*_. We begin by finding the distribution of the minimum number of burst events *N* required for *x*(*t*) to reach the threshold *X*. In particular,


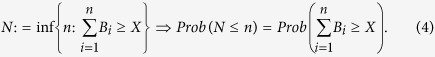


Given a specific form for the distribution of *B*_*i*_, the corresponding distribution for *N* can be obtained using equation (4). For example, if *B*_*i*_ is geometrically distributed, then the probability mass function of *N* is given by


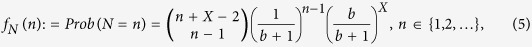


where *b* represents the mean burst size^27^,^28^.

Having determined the number of bursts needed for cell division, we next focus on the timing of burst events. Let *T*_*n*_ represent the time at which *n*^*th*^ burst event takes place. If the burst arrival rate in equation (1) were constant, then the time intervals between bursts would be exponentially distributed, resulting in an Erlang distribution for *T*_*n*_. However, in our case this rate is time varying (due to dependence on cell volume), the arrival of bursts is an inhomogeneous Poisson process. Employing the distribution for the timing of the *n*^*th*^ event, and using the fact that *FPT* is same as the time at which the *N*^*th*^ burst event occurs, the probability density function of *FPT* is obtained as


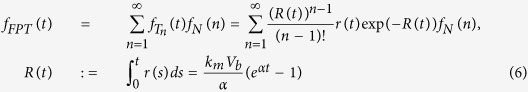


(see SI, section S2). One can note that *f*_*FPT*_(*t*) is dependent on the newborn cell size *V*_*b*_ through the function *R*(*t*).

**Figure 2:**
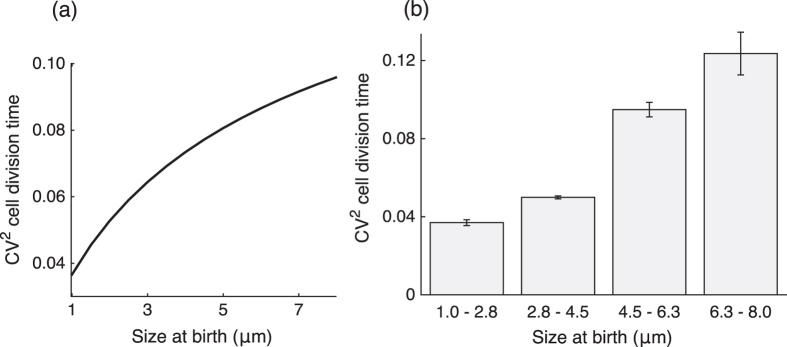
Both model prediction and data show increase in the noise in timing as newborn cell size increases. **(a)** Model prediction for noise (coefficient of variation squared, *CV*^2^) in division time as computed numerically using equation (6). The model parameters used are: transcription rate *k*_*m*_ = 0.13 min^−1^, threshold *X* = 65 molecules, growth rate *α* = 0.03 min^−1^, and mean burst size *b* = 5 molecules. The distribution of protein burst size *B*_*i*_ is assumed to be geometric. For details on how these parameter values were estimated, see SI, section S6. **(b)** Experimental data from^1^ for *Escherichia coli MG1655* also shows increase in cell division time noise as newborn cell size increases. Single-cell data was categorized in one of the four bins (1–2.8 *μm*, 2.8–4.5 *μm*, 4.5–6.3 *μm*, and 6.3–8 *μm*) depending upon newborn cell sizes. *CV*^2^ of division time with 95% confidence interval (using bootstrapping) for each bin is shown (more details in SI, section S6).

This *FPT* distribution qualitatively emulates the experimental observations that the mean cell division time decreases with increasing cell size at birth (see SI, section S6). Intuitively, a larger newborn cell expresses the protein at a higher rate as compared to a smaller cell. Hence, the time taken by the protein to reach the prescribed molecular threshold is shorter in larger cells. Analysis of equation (6) also predicts that the noise (quantified using the coefficient of variation squared, *CV*^2^) in cell-division timing increases with increasing *V*_*b*_, and we confirmed this behavior from published data (Fig. 2). The noise behavior can be understood from the fact that a small newborn cell requires more time for cell division. This allows for efficient time averaging of the underlying bursty process resulting in lower stochasticity in *FPT*.

### Distribution of the volume added between divisions

**Figure 3:**
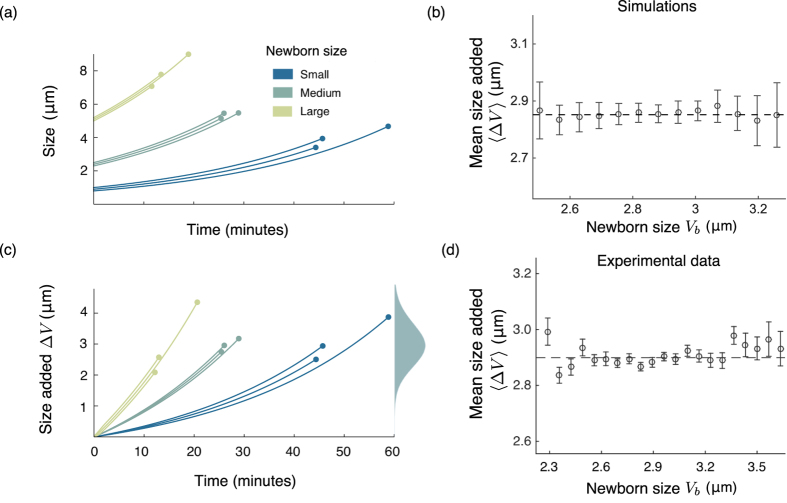
The proposed mechanism results in added cell size distribution being independent of the newborn cell size. **(a)** The cell volume grows exponentially (shown for three different newborn cell sizes) until the timekeeper protein reaches a critical threshold. **(b)** The size added to the newborn cell size also grows exponentially until division takes place. For three different newborn cell sizes, the distribution of the the added volume comes out to be same. **(c)** The added size generated via simulations is plotted against the newborn cell size in range 2–3.5 *μm* for 10,000 cells. The cells are further binned in 13 uniformly spaced bins (number of cells per bin >100). The dashed line shows the mean of the added volume, which is independent of the newborn cell size. **(d)** Data from^6^ showing the added size versus newborn cell size for *Escherichia coli NCM3722* grown in Glucose as carbon source. Cells were categorized into bins according to their newborn cell size (number of cells per bin >100). For each bin, the circle shows mean of the added size whereas the error bar represents the standard deviation of the added size. It can be seen that the mean added cell size (shown by dashed line) is independent of the newborn cell size (also see Fig. 2D in ref. 6).

Having derived the distribution for the cell-division time (*FPT*), we determine the volume added by a single cell from birth to division (denoted by Δ*V*). Since volume grows exponentially, Δ*V* is related to *FPT* as Δ*V* = *V*_*b*_(*e*^*αFPT*^−1). Using the distribution of *FPT* from equation (6) yields the following probability density function for Δ*V*


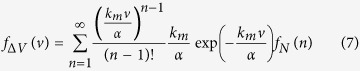


(see SI, section S3). One striking observation is that *f*_Δ*V*_ (*v*) is independent of the initial volume *V*_*b*_ (as illustrated in Fig. 3). This is in agreement with experimental observations that the histograms of the added volume for different newborn cell sizes are statistically identical^6^. Next, we investigate how statistical moments of Δ*V* depend on model parameters, in particular, the growth rate *α*.

### Mean volume added between divisions

Using equation (7), the average volume added is obtained as


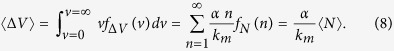


Here 〈*N*〉 represents the mean number of protein burst events from cell birth to division, which depends on the threshold *X* and the form of the burst size distribution. For example, if the protein bursts *B*_*i*_ are geometrically distributed with mean *b*, then using equation (5)


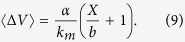


These formulas reveal a linear dependence of Δ*V* on *α*, in agreement with data from *Pseudomonas aeruginosa*^7^. It turns out that the dependency of Δ*V* on *α* can vary among bacterial species. For instance, *Caulobacter crescentus* exhibits an added volume independent of *α*, whereas this relationship is thought to be exponential in case of *Escherichia coli*^5^,^6^. Studies connecting cellular growth rates to gene expression parameters have shown that *α* primarily affects the transcription rate, with mRNA translation and stability being largely invariant across growth conditions^29^,^30^. Thus, if the transcription rate *k*_*m*_ is a linear function of *α*, then Δ*V* becomes independent of *α*. Next, we discuss a slightly different model formulation that results in exponential dependency of Δ*V* on *α*.

So far we have considered that the timekeeper protein observes time from cell birth to division. In principle, the timekeeping could be for some other important event in the cell cycle. Consider a scenario where the initiation of DNA replication takes place when sufficient timekeeper protein has accumulated per origin of replication^10^,^31^,^32^,^33^. The corresponding division event is assumed to occur with a constant delay of *T* after an initiation. The delay *T* here is the *C* + *D* period, where *C* represents the time to replicate the DNA and *D* denotes the time between DNA replication and division^34^,^35^. As growing bacterial cells are known to regulate the number of DNA replication forks as a function of growth rate, we assume that the threshold for the timekeeper proteins changes accordingly. More specifically, if there are *θ* origins of replication, the number of timekeeper protein molecules required to be accumulated for the next initiation event are *θX*. The above assumption is consistent with the understanding that all origins of replication fire almost synchronously^36^,^37^. Further, the timekeeper molecules are assumed to get degraded (deactivated) after initiation and a new set of timekeeper molecules are produced for the next initiation. Upon a division event between two successive initiations, the partitioning errors in the timekeeper protein are assumed to be negligible.

In this alternative formulation, the average volume added between two consecutive initiation events for each origin of replication is approximately same as Δ*V* obtained in equation (9) (see SI, section S3). Moreover, the average volume added between successive division events is now given by^33^


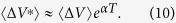


Recall from equation (9) that 〈Δ*V*〉 depends linearly on *α*. Thus, the expression in equation (10) suggests two different regimes of how 〈ΔV^*^〉 depends upon *α*. For small values of *α*, *α* exp (*αT*) ≈ *α*, i.e., the mean added volume increases linearly with the growth rate. In the regime where *α* is large, the exponential term dominates. This implies that if *α* is small, it may not be possible to distinguish whether the underlying mechanism accounts for volume added between two division events or two initiation events as the data will show a linear dependence of the average added volume with changes in *α*^7^. Notice that a pure exponential relationship between 〈ΔV^*^〉 and *α* can also be obtained if *k*_*m*_ is a linearly increasing function of *α*. For this particular case, the volume accounted by each origin of replication 〈Δ*V*〉 becomes invariant of the growth rate, consistent with previous works^33^,^38^. In summary, depending on the underlying assumptions, the model captures a variety of relationships between the average volume added from cell birth to division and *α*.

It is noteworthy that in the above setup, dependency of the time *T* = *C* + *D* on growth rate or cell size has been neglected even though there is evidence that *D* usually depends upon both growth rate and cell size^39^. We have done so for simplicity as incorporating this would not change the fact that an exponential dependency can be generated between Δ*V* and *α* by having the protein account for two other events in the cell cycle. We next investigate higher order moments of Δ*V* in the original model formulation, where the timekeeper protein accounts for timing between division events.

### Higher order moments of added volume

**Figure 4:**
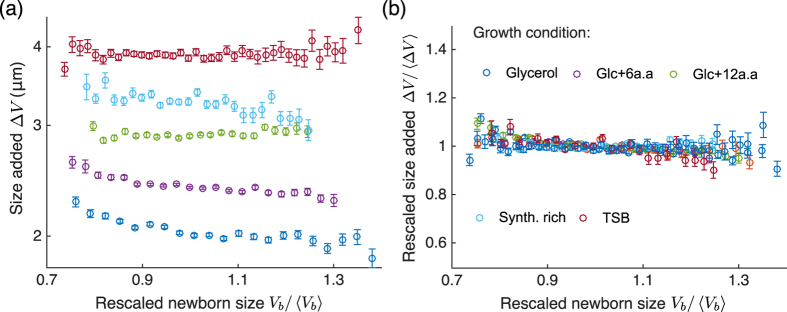
Collapse of added cell size in different growth conditions upon rescaling by respective mean values. **(a)** Using data from^6^ for *Escherichia coli NCM3722*, the added size is plotted versus the newborn cell size for different growth conditions. The mean added size (shown by circles) for each growth condition is different for a given newborn cell size. Cells were categorized into bins according to their newborn cell sizes (number of cells per bin >100). The error bars represent the standard deviation of the added volume of cells in each bin. **(b)** The added size data for different growth conditions collapse upon rescaling them by their means in the respective growth conditions (also see Fig. 2D in ref. 6).

We can use the distribution of Δ*V* computed in equation (7) to get insights into its higher-order statistics such as coefficient of variation squared 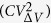 and skewness (*skew*_Δ*V*_). For example, when the protein production occurs in geometric bursts


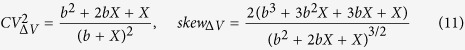


(see SI, section S3). Note that Δ*V* is always positively skewed, consistent with previous understanding^9^. Moreover, both *CV*^2^ and skewness are independent of the growth rate *α*. It turns out an even more general property is true: an appropriately scaled *j*^*th*^ order moment of Δ*V*, i.e., 〈Δ*V*^*j*^〉/〈Δ*V*〉^*j*^ is independent of *α*, in spite of the underlying distribution of the burst size. This arises from the fact that the distribution of Δ*V* can be written in the following form


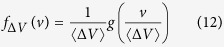


for some function *g* (see SI, section S3). This form implies that *f*_Δ*V*_(*v*) is scale invariant: the shape of the distribution across different growth rates is essentially the same, and a single parameter 〈Δ*V*〉 is sufficient to characterize the distribution of Δ*V*^40^. This property was seen in experiments^6^,^11^,^41^, where the histograms for Δ*V*/〈Δ*V*〉 in different growth conditions collapse upon each other (Fig. 4).

Interestingly, the above invariance property is not limited to the distribution of the added volume Δ*V*. As the distributions of the cell size at birth, and cell size at division are generated by weighted sums of random variables drawn from the distribution of Δ*V*, they naturally inherit the scale-invariance property^6^ (see SI, section S4). Furthermore, the distribution of the cell-division time also has the scale invariance property (see SI, section S5), which is in agreement with previous works^42^,^43^.

## Discussion

It is now well understood that several prokaryotes, such as, *Escherichia coli, Caulobacter crescentus, Bacillus subtilis and Pseudomonas aeruginosa* employ an adder mechanism for size homeostasis^5^,^6^,^7^,^8^. In this work, we studied a simple molecular mechanism for realizing the adder principle that consists of a timekeeper protein expressed at a rate proportional to cell volume up to a critical threshold. Our work shows that stochastic expression of this protein is sufficient to explain the statistical properties of the cell-division time and the size added from cell birth to division. Key model insights are as follows:

- Distribution of the volume added from birth to division is independent of the newborn cell volume, a hallmark of the adder principle (Fig. 3).
- The distributions of key quantities such as the added volume, division time, volume at birth and division are scale invariant.
- The noise in cell-division time increases with increasing newborn cell size (Fig. 2).

An important point to note is that if variation in Δ*V* is indeed a result of noisy gene expression, then Δ*V* for successive cell-cycles should be independent. Indeed, data shows a weak correlation between the volume added for mother and daughter cells^5^,^6^. This result also argues that extrinsic fluctuations in parameters that exhibit strong memory between mother and daughter cells cannot account for the statistical fluctuations in Δ*V*.

A natural question that arises at this point is whether there are known proteins which mimic the dynamics of the timekeeper protein. Among many proteins involved in the cell cycle control, prominent candidates for the timekeeper protein are FtsZ and DnaA. More specifically if the constant volume addition is considered between division to division, FtsZ could be acting as the proposed timekeeper protein^44^,^45^,^46^,^47^,^48^. It has been proposed that the accumulation of FtsZ up to a critical level is required for cell division^49^,^50^,^51^. Interestingly, in case of *Caulobacter crescentus*, FtsZ is synthesized in a cell cycle dependent manner and its degradation rate increases after the initiation of cell division^44^. However, in case of *Escherichia coli*, its concentration remains constant throughout the cell cycle^52^. It is possible that in the former case FtsZ molecules realize the timekeeping whereas in latter case it is realized by the assembly dynamics of the *Z*-ring^50^. Further, in the other possibility when the constant volume is added between two initiation events, DnaA could behave as the timekeeper^48^,^53^,^54^. In this case, concentration of DnaA remains constant and initiation is proposed to occur when a critical number (around 20) of DnaA-ATP (active form of DnaA) molecules are available. After initiation these molecules are converted to DnaA-ADP (inactive form)^55^. While more systematic studies are warranted to ascertain roles of these proteins, their dynamics broadly satisfies the requirements of the hypothesized timekeeper protein. It should also be noted that the exact molecular implementations vary between species and the timekeeper protein is possibly only responsible for a coarse tuning of the cell division process. More accurate descriptions of the process will also require to account for feedbacks between important cell-cycle events. For example, it is well-known that FtsZ does not proceed with Z-ring formation until DNA replication has faithfully taken place. If this condition is not met, the adder principle would probably be overridden^5^.

**Figure 5:**
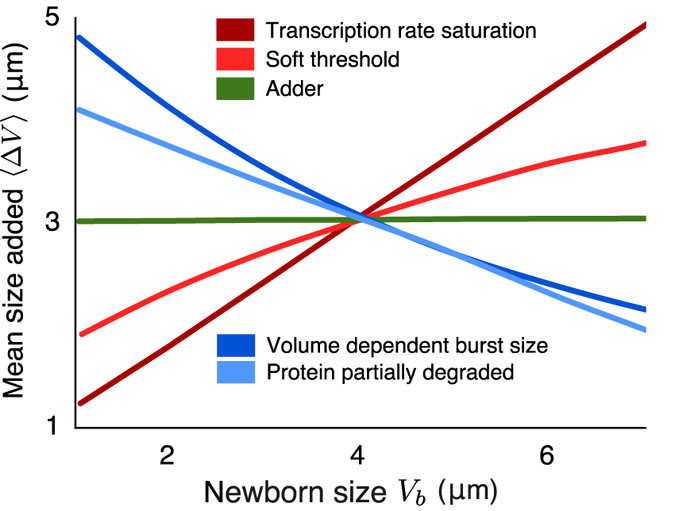
Molecular mechanisms resulting in deviations from the adder principle. The added cell size (Δ*V*) versus newborn cell size (*V*_*b*_) is plotted for four different mechanisms. From these hypothesized mechanisms, partial degradation of the time-keeper protein, and volume-dependent burst size result in negative correlation between the added volume and newborn cell size. In contrast, the other two mechanisms (transcription rate being a saturating function of cell volume, and soft threshold for triggering of the event) result in a positive correlation. For each of these mechanisms, the plots are generated from 10,000 realizations of a cell cycle starting with a newborn cell size *V*_*b*_ in the interval [1, 7]*μm*, computing the volume added since birth for each of them, and then taking average. The simulations were done using algorithm proposed in ref. 58.

Recent work in *Escherichia coli* has observed deviations from the adder principle for some strains under some growth conditions^56^. Unlike the adder principle, here Δ*V* does not exhibit constancy with respect to newborn cell size and instead shows non-zero correlations^56^,^57^. For instance, the strain *MG*4100 shows slight positive correlations between added volume and newborn cell size when grown at 25 °C and 27 °C. Interestingly, the same strain also shows negative correlations between the added volume and newborn cell size at 37 °C^56^. While it is not clear as to why these deviations are seen in some conditions, there could be several mechanisms that result in such deviations in our proposed model. These include: i) the timekeeper protein does not degrade fully upon division and the remaining proteins are divided in the daughter cells; ii) an indirect feedback from the cell volume to the mRNA translation rate making protein burst sizes volume-dependent; iii) saturation in the transcription rate 
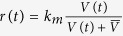
 at high cell volume; iv) a soft threshold for the protein level in equation (3), where cell-division is triggered even before attainment of a molecular threshold. Preliminary stochastic simulations show that in all these cases Δ*V* becomes dependent on the newborn cell size. Specifically, mechanisms (i) and (ii) show negative correlations between added volume and newborn cell size whereas mechanisms (iii) and (iv) show positive correlations (Fig. 5). As the strain *MG*4100 shows both negative and positive correlations as temperature is varied, we speculate that a combination of mechanisms that generated positive and negative deviations could be at play. As the temperature is varied, one of these deviations could become dominant. Clearly, a more systematic analysis with computations of the cell-division time and volume added distributions is warranted in these cases. It will be interesting to see if the statistical properties of Δ*V* and the cell-division time contain signatures to discriminate between alternative models and provide insights into the regulatory mechanisms that drive deviations from the adder principle.

## Additional Information

**How to cite this article**: Ghusinga, K. R. *et al*. A mechanistic stochastic framework for regulating bacterial cell division. *Sci. Rep*. **6**, 30229; doi: 10.1038/srep30229 (2016).

## Supplementary Material

Supplementary Information

## Figures and Tables

**Figure 1:**
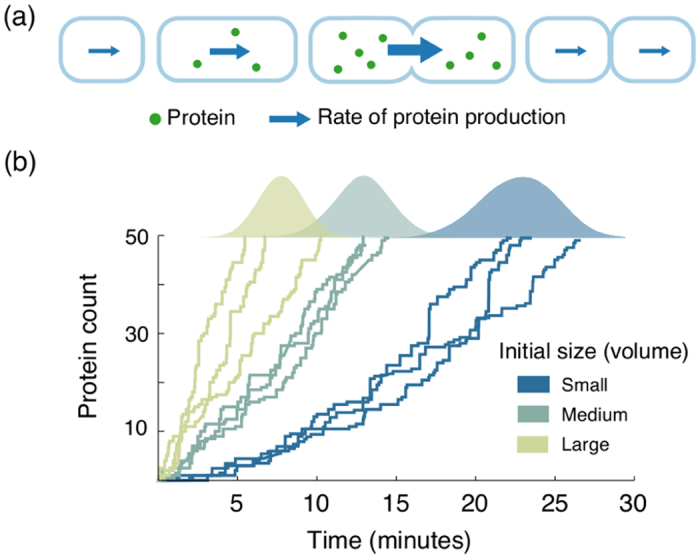
Proposed molecular mechanism to realize adder principle of cell size control. **(a)** An exponentially growing rod-shaped cell starts synthesizing a timekeeper protein after its birth. The production rate of the protein scales with the cell size (volume). When the protein’s copy number attains a certain level, the cell divides and the protein is degraded. **(b)** Stochastic evolution of the protein copy numbers is shown for cells of three different sizes at birth. The threshold for triggering cell division is assumed to be 50 molecules. The distribution of the first-passage time (generated via 1,000 Monte Carlo realizations) for each newborn cell volume is shown above the three corresponding trajectories. The first-passage time distribution depends upon the newborn cell size: on average, the protein in a smaller cell takes more time to reach the threshold as compared to the protein in a larger cell.
