# Supplementary material for: A mechanistic stochastic framework for regulating bacterial cell division
Source: Sci Rep. 2016 Jul 26;6:30229. doi: 10.1038/srep30229 (PMC4960620; doi:10.1038/srep30229)
Supplement: Supplementary Information [file srep30229-s1.pdf]

Supplementary Information for
"A mechanistic stochastic framework for bacterial cell-division timing"

Khem Raj Ghusinga, Cesar A. Vargas-Garcia, Abhyudai Singh

S1 Theoretical constraints on the molecular mechanism underlying the adder principle

The adder principle states that a cell adds a constant volume between birth to division notwithstanding its size at birth [1–3]. In order to investigate what molecular mechanisms could realize this behavior, we consider a hybrid system framework shown in Fig. S1. The model here consists of a state variable, ΔV, which denotes the volume added to a cell's volume at birth. The dynamics of ΔV is given by the following ordinary differential equation:

$$\dot{\Delta V} = \alpha(\Delta V + V_b), \tag{S1.1}$$

where V_b is the volume of a cell at birth, and α represents the growth rate. Furthermore, we assume that the division occurs at an added volume dependent rate $h(\Delta V)$. Upon division, the added volume ΔV resets to zero whereas the cell volume at birth resets to $(V_b + \Delta V)/2$.

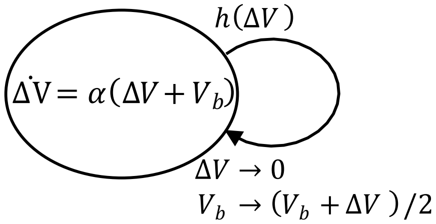

Figure S1: *Description of the cell division process as a stochastic hybrid system.* The added volume ΔV evolves as per a deterministic dynamics until the division event takes place. The hazard rate for division is $h(\Delta V)$. Upon division, the added volume ΔV and the cell volume at birth V_b reset to 0 and $(V_b + \Delta V)/2$, respectively.

Using the infinitismal generator of a stochastic hybrid system, one can write the time evolution of expected added volume $\langle \Delta V \rangle$ as [4]:

$$\frac{d}{dt}\langle \Delta V \rangle = \Big\langle \alpha(\Delta V + V_b) - h(\Delta V)\Delta V \Big\rangle. \tag{S1.2}$$

Using an mean-field approximation, the above equation can be written as

$$\frac{d}{dt}\langle \Delta V \rangle \approx \alpha(\langle \Delta V \rangle + V_b) - h(\Delta V)\langle \Delta V \rangle, \tag{S1.3}$$

In steady state, a solution of equation (S1.3) which has $\langle \Delta V \rangle$ independent of V_b is only possible if the hazard rate has following form:

$$h(\Delta V) = 0 \quad \text{for} \quad \Delta V < \overline{\Delta V}, \tag{S1.4}$$

$$h(\Delta V) = \infty \quad \text{for} \quad \Delta V > \overline{\Delta V}, \tag{S1.5}$$

where $\overline{\Delta V}$ denotes the volume that a cell attempts to add. In other words, any mechanism which actively senses the added volume has to trigger the division the moment it reaches the prescribed threshold. In the main text, we propose a timekeeper protein to trigger the division. Assuming a deterministic production of the timekeeper protein, results here imply that the division rate follows

$$h(x) = 0 \quad \text{for} \quad x < X, \tag{S1.6}$$

$$h(x) = \infty \quad \text{for} \quad x > X, \tag{S1.7}$$

where x represents the protein level, and X is the prescribed protein copy number threshold for division. This constraint on the division rate was realized by computing the division time as the first-passage time in the main text. Also note that as the main paper describes, the timekeeper protein based mechanism led to realization of the adder model in distribution sense. Therefore the theoretical constraint in equations (S1.6)-(S1.7) is both necessary and sufficient to realize the adder principle of cell size control.

S2 Distribution of FPT given cell volume at birth

As described in the main text (equation (4)), the distribution of the minimum number of burst (transcription) events N required for $x(t)$ to reach the threshold X is computed by using

$$Prob\,(N \le n) = Prob\left(\sum_{i=1}^{n} B_i \ge X\right). \tag{S2.1}$$

Given a specific form for the distribution of B_i, the corresponding distribution for N can be obtained using equation (S2.1) (two specific examples are discussed later in this section).

Having determined the number of bursts needed for cell division, we next focus on the timing of burst events which is determined by the burst arrival rate. Here since the burst arrival rate is time varying (due to dependence on cell volume), the arrival process an inhomogeneous Poisson process. Prior work on inhomogeneous Poisson processes has shown that the distribution for the timing of the nth event is given by [5,6]

$$f_{T_n}(t) = \frac{(R(t))^{n-1}}{(n-1)!} r(t) \exp(-R(t)), \quad R(t) := \int_0^t r(s)ds = \frac{k_m V_0}{\alpha}\left(e^{\alpha t} - 1\right). \tag{S2.2}$$

Note that FPT is the same as the time at which the Nth burst event occurs. Thus, the probability density function of FPT is obtained as

$$f_{FPT}(t) = \sum_{n=1}^{\infty} f_{T_n}(t) f_N(n) = \sum_{n=1}^{\infty} \frac{(R(t))^{n-1}}{(n-1)!} r(t) \exp(-R(t)) f_N(n) \tag{S2.3}$$

and is dependent on the newborn cell size V_0 through the function $R(t)$ defined in (S2.2). Next, using the relation in (S2.1), we quantify the distribution N from the distribution of protein burst size B_i.

Probability mass function of N for common burst size distributions

We present the form of distribution of N for two relevant cases here: when the protein burst size is one with probability one, and when the protein burst size is geometric [7–12]. For the case when the burst size B_i is one with probability one, *exactly* X events are required for the protein level $x(t)$ to reach X for the first time. That is, we have

$$f_N(n) = \delta(n-X), \tag{S2.4}$$

where $\delta(n-X)$ is the Kronecker's delta which is one when $n = X$ and zero otherwise.

When the burst size B_i follows a geometric distribution [7–12], the calculation of the minimum number of transcription events N for this distribution has been previously done in our works [13,14]. The probability mass function of N is given by

$$f_N(n) = \binom{n+X-2}{n-1}\left(\frac{1}{b+1}\right)^{n-1}\left(\frac{b}{b+1}\right)^{X}. \tag{S2.5}$$

Here b represents the mean protein burst size. Further, the first three statistical moments of N given by the above probability mass function are

$$\langle N \rangle = \frac{X}{b} + 1, \tag{S2.6}$$

$$\left\langle N^2 \right\rangle = \frac{b^2 + 3bX + X + X^2}{b^2}, \tag{S2.7}$$

$$\left\langle N^3 \right\rangle = \frac{b^3 + 7b^2X + 6bX(X+1) + X\left(X^2 + 3X + 2\right)}{b^3}. \tag{S2.8}$$

These formulas are used in the next section to compute the moments of the volume added between birth to division.

S3 Distribution of volume added ΔV

Let V_b denote the volume of a newborn cell. Assuming birth of a cell at $t = 0$, the volume after at a time t is given by $V(t) = V_b \exp(\alpha t)$. We assume that the cell divides at the first-passage time whose distribution is given by equation (S2.3). Representing the volume added to the cell's volume at birth until division by ΔV, we have

$$\Delta V = V_b\left(e^{\alpha FPT} - 1\right). \tag{S3.1}$$

The cumulative distribution function of ΔV can be computed as

$$Prob\{\Delta V \leq v\} = Prob\left\{V_b\left(e^{\alpha FPT} - 1\right) \leq v\right\} \tag{S3.2}$$

$$= Prob\left\{FPT \leq \frac{1}{\alpha}\ln\left(\frac{v}{V_b} + 1\right)\right\} \tag{S3.3}$$

$$= \int_0^{\frac{1}{\alpha}\ln\left(\frac{v}{V_b}+1\right)} f_{FPT}(t)dt. \tag{S3.4}$$

Differentiating the above expression results in the probability density function of ΔV as follows

$$f_{\Delta V}(v) = \frac{d}{dv}\left(Prob\{\Delta V \leq v\}\right) \tag{S3.5}$$

$$= \frac{d}{dv}\int_0^{\frac{1}{\alpha}\ln\left(\frac{v}{V_b}+1\right)} f_{FPT}(t)dt \tag{S3.6}$$

$$= f_{FPT}\left(\frac{1}{\alpha}\ln\left(\frac{v}{V_b}+1\right)\right)\frac{d}{dv}\left(\frac{1}{\alpha}\ln\left(\frac{v}{V_b}+1\right)\right). \tag{S3.7}$$

Note that $\frac{d}{dv}\left(\frac{1}{\alpha}\ln\left(\frac{v}{V_b}+1\right)\right)=\frac{1}{\alpha\left(V_b+v\right)}$. Also

$$R\left(\frac{1}{\alpha}\ln\left(\frac{v}{V_b}+1\right)\right)=\frac{k_m v}{\alpha},\quad r\left(\frac{1}{\alpha}\ln\left(\frac{v}{V_b}+1\right)\right)=k_m\left(v+V_b\right).$$

Hence we can write the probability density of ΔV as

$$f_{\Delta V}(v)=\frac{1}{\alpha\left(V_b+v\right)}\sum_{n=1}^{\infty}\frac{\left(\frac{k_m v}{\alpha}\right)^{n-1}}{(n-1)!}k_m\left(V_b+v\right)\exp\left(-\frac{k_m v}{\alpha}\right)f_N(n) \tag{S3.8}$$

$$=\sum_{n=1}^{\infty}\frac{\left(\frac{k_m v}{\alpha}\right)^{n-1}}{(n-1)!}\frac{k_m}{\alpha}\exp\left(-\frac{k_m v}{\alpha}\right)f_N(n). \tag{S3.9}$$

Notice that this distribution is an Erlang distribution conditioned to the random variable N.

Moments of ΔV

Mean ΔV Since the distribution of ΔV is conditional Erlang, we have the following expression for mean ΔV.

$$\langle\Delta V\rangle=\sum_{n=1}^{\infty}\frac{n}{k_m/\alpha}f_N(n)=\frac{\alpha}{k_m}\langle N\rangle. \tag{S3.10}$$

Second order moment The second order moment of ΔV is given by

$$\left\langle\Delta V^2\right\rangle=\sum_{n=1}^{\infty}\frac{n^2+n}{\left(k_m/\alpha\right)^2}f_N(n)=\frac{\alpha^2}{k_m^2}\left(\left\langle N^2\right\rangle+\langle N\rangle\right). \tag{S3.11}$$

Third order moment The third order moment of ΔV is given by

$$\left\langle\Delta V^3\right\rangle=\sum_{n=1}^{\infty}\frac{n^3+3n^2+2n}{\left(k_m/\alpha\right)^3}f_N(n)=\frac{\alpha^3}{k_m^3}\left(\left\langle N^3\right\rangle+3\left\langle N^2\right\rangle+2\langle N\rangle\right). \tag{S3.12}$$

When the burst size is one with probability one, we have $N=X$ with probability one. The formulas of mean, CV^2 and skewness of ΔV simplify to

$$\langle\Delta V\rangle=\frac{\alpha X}{k_m},\quad CV_{\Delta V}^2=\frac{1}{X},\quad skew_{\Delta V}=\frac{2}{\sqrt{X}}. \tag{S3.13}$$

When the burst size is geometric, we employ the expressions of moments of N from section S1 to get the following expressions:

$$\langle\Delta V\rangle=\frac{\alpha}{k_m}\left(\frac{X}{b}+1\right), \tag{S3.14}$$

$$CV_{\Delta V}^2=\frac{var(\Delta V)}{\langle\Delta V\rangle^2}=\frac{b^2+2bX+X}{(b+X)^2}, \tag{S3.15}$$

$$skew(\Delta V)=\frac{2\left(b^3+3b^2X+3bX+X\right)}{\left(b^2+2bX+X\right)^{3/2}}. \tag{S3.16}$$

We also note that that the skewness of ΔV is positive in both cases considered above which is consistent with previous results [1].

Scale invariance of the distribution of ΔV

It has been shown in [3] that the distributions of the added volume ΔV in different growth conditions collapse when rescaled by respective $\langle \Delta V \rangle$. Mathematically, we want to show that the probability density function $f_{\Delta V}(v)$ has the following form:

$$f_{\Delta V}(v) = \frac{1}{\langle \Delta V \rangle} g\left(\frac{v}{\langle \Delta V \rangle}\right), \tag{S3.17}$$

where $g(.)$ is an arbitrary normalized function [3, Supplementary Information equation 36]. For the distribution in equation (S3.9), we consider

$$g(w) = \sum_{n=1}^{\infty} \frac{(w\langle N \rangle)^{n-1}}{(n-1)!} \langle N \rangle \exp\left(-w\langle N \rangle\right) f_N(n), \tag{S3.18}$$

where $\langle N \rangle$ is the expected value of the minimum number of transcription events required for the protein to cross the threshold X. Also, it is related with $\langle \Delta V \rangle$ as described in equation (S3.10).

For the function g given in equation (S3.18), we have

$$\frac{1}{\langle \Delta V \rangle} g\left(\frac{v}{\langle \Delta V \rangle}\right) = \frac{1}{\langle \Delta V \rangle} \sum_{n=1}^{\infty} \frac{\left(\frac{v\langle N \rangle}{\langle \Delta V \rangle}\right)^{n-1}}{(n-1)!} \langle N \rangle \exp\left(-\frac{v\langle N \rangle}{\langle \Delta V \rangle}\right) f_N(n) \tag{S3.19}$$

$$= \sum_{n=1}^{\infty} \frac{\left(\frac{k_m v}{\alpha}\right)^{n-1}}{(n-1)!} \frac{k_m}{\alpha} \exp\left(-\frac{k_m v}{\alpha}\right) f_N(n) \tag{S3.20}$$

$$= f_{\Delta V}(v). \tag{S3.21}$$

This establishes the scale invariance of the distribution $f_{\Delta V}(v)$.

One consequence of the scale invariance property of $f_{\Delta V}(v)$ is that the normalized moments $\langle \Delta V^j \rangle / \langle \Delta V \rangle^j$ are independent of the growth conditions [3, Supplementary Information]. This can be checked as follows.

The jth order conditional moment of ΔV (conditioned with respect to N) is given by j^{th} order moment of an Erlang distribution. Thus

$$\left\langle \Delta V^j | N = n \right\rangle = \left(\frac{\alpha}{k_m}\right)^j (n(n+1)(n+2)\cdots(n+j-1)) \tag{S3.22}$$

$$\implies \left\langle \Delta V^j \right\rangle = \left(\frac{\alpha}{k_m}\right)^j \langle N(N+1)(N+2)\cdots(N+j-1)\rangle. \tag{S3.23}$$

Therefore using equation (S3.10), we have

$$\frac{\left\langle \Delta V^j \right\rangle}{\langle \Delta V \rangle^j} = \frac{\langle (N(N+1)(N+2)\cdots(N+j-1))\rangle}{\langle N \rangle^j} \tag{S3.24}$$

which is independent of the growth rate.

This fact can be used to show that statistical measures such as noise (CV^2) and skewness are independent of the growth rate. Take CV^2 for instance. It is defined as

$$CV_{\Delta V}^2 = \frac{\left\langle \Delta V^2 \right\rangle}{\langle \Delta V \rangle^2} - 1. \tag{S3.25}$$

By the scale invariance property, $\langle \Delta V^2 \rangle / \langle \Delta V \rangle^2$ is independent of the growth rate α. Thus, the noise CV^2 is also independent of α. Similarly, skewness is given by

$$skew_{\Delta V} = \frac{\langle \Delta V^3 \rangle - 3\langle \Delta V \rangle var(\Delta V) - \langle \Delta V \rangle^3}{(var(\Delta V))^{3/2}} \tag{S3.26}$$

$$= \frac{\frac{\langle \Delta V^3 \rangle}{\langle \Delta V \rangle^3} - 3\left(\frac{\langle \Delta V^2 \rangle}{\langle \Delta V \rangle^2} - 1\right) - 1}{\left(\frac{\langle \Delta V^2 \rangle}{\langle \Delta V \rangle^2} - 1\right)^{3/2}}, \tag{S3.27}$$

which is again independent of α by the scale invariance property.

S3-a Model analysis when the timekeeper protein is accumulated between two initiation events

In the previous model formulation, we consider that the timekeeper protein is accumulated between cell birth to division. Here we analyze the model when the accumulation is between two other events in cell cycle (namely initiation of DNA replication), and the corresponding division for an initiation event takes place after a constant time delay T.

Assume a cell with volume V_{init} right after an initiation event. At this point, the timekeeper molecules are degraded (or deactivated), and new set of timekeeper proteins are synthesized (or activated) for the next initiation event. These proteins are synthesized at a rate $k_m V_{init} \exp(\alpha t)$, and the threshold required to be achieved for the next initiation events is θX where θ is the number of origins of replication right after the previous initiation event. For time being, we will ignore any division events until the next initiation event.

Following the calculations used to derive equation (S3.9), one can write the distribution of the volume added until the next initiation event as

$$f_{\Delta V}(v) = \sum_{n=1}^{\infty} \frac{\left(\frac{k_m v}{\alpha}\right)^{n-1}}{(n-1)!} \frac{k_m}{\alpha} \exp\left(-\frac{k_m v}{\alpha}\right) f_N(n), \tag{S3.28}$$

where the distribution of the number of transcription events $f_N(n)$ is now given by

$$f_N(n) = \delta(n - \theta X) \qquad \text{(for deterministic burst)}, \tag{S3.29}$$

$$f_N(n) = \binom{n + \theta X - 2}{n-1} \left(\frac{1}{b+1}\right)^{n-1} \left(\frac{b}{b+1}\right)^{\theta X} \qquad \text{(for geometric burst)}. \tag{S3.30}$$

One can write the average volume per origin of replication added between the initiation events are

$$\langle \Delta V \rangle = \frac{1}{\theta} \frac{\alpha \theta X}{k_m} = \frac{\alpha X}{k_m} \qquad \text{(for deterministic burst)}, \tag{S3.31}$$

$$\langle \Delta V \rangle = \frac{1}{\theta} \frac{\alpha}{k_m} \left(\frac{\theta X}{b} + 1\right) = \frac{\alpha}{k_m} \left(\frac{X}{b} + \frac{1}{\theta}\right) \qquad \text{(for geometric burst)}. \tag{S3.32}$$

Note that for a deterministic burst, the average volume added per origin of replication is same as the volume added between birth and division for the previous model. For geometric burst case, the added volumes are approximately same.

Recall that we had ignored division events between two initiation events for above analysis. If there is a division event between two initiation events, both the origins of replication and the timekeeper proteins

accumulated since previous initiation event can be assumed to divide equally into the daughter cells. Consequently, for each daughter cell, the threshold for next initiation event will become approximately half of what it was for the mother cell. Thus, one can ignore the division event and consider the total volume as one cell until the next initiation event takes place. One can, of course, expect stochastic effects in the partitioning of protein molecules but inclusion of them should not change the average behavior discussed here.

S4 Distribution of newborn cell size V_b

In this section, we determine the distribution of newborn cell size V_b, and discuss its scale invariance property. For this purpose, we consider a newborn cell whose volume is V_0 and observe its volume after subsequent cell cycles. Let V_p denote the cell volume after $p \geq 1$ cell cycles, then we have

$$V_p = 2^{-p} V_0 + 2^{-p} \sum_{i=1}^{p} 2^{i-1} \Delta V_i, \tag{S4.1}$$

where ΔV_i denotes the volume added during the ith cell cycle. The random variables $\Delta V_i,\ i \in \{1, 2, \ldots, p\}$ are independent and identically distributed, and their distribution is same as that of ΔV as given by equation (S3.9). At each division, the cell is assumed to divide symmetrically.

The steady-state distribution of V_p, i.e., when $p \to \infty$ would give the distribution of V_b. Note that the contribution from the initial cell volume V_0 in equation (S4.1) decays exponentially. Therefore, we can make a first approximation as

$$V_p \approx 2^{-p} \sum_{i=1}^{p} 2^{i-1} \Delta V_i. \tag{S4.2}$$

In essence, V_p is a weighted sum of independent and identically distributed random variables ΔV_i. As the following holds for the weights in above sum in equation (S4.2)

$$\sum_{i=1}^{p} (2^{i-p-1})^m = \frac{(1-2^{-p})^m}{2^m - 1} < 1, \quad \forall m \in \{1, 2, 3, \ldots\}, \tag{S4.3}$$

one can use standard probability theory arguments to show that the distribution of V_p converges. In fact, for the weights we have in equation (S4.2), the mean and variance of V_p respectively converge to $\langle \Delta V \rangle$, and $\left(\langle \Delta V^2 \rangle - \langle \Delta V \rangle^2 \right)/3$ as $p \to \infty$.

Furthermore, we can also find the expression of the probability density function of V_p by making use of probability density function of random variables ΔV_i in equation (S3.9). It is easy to show that the random variables $\overline{\Delta V}_i := 2^{i-p-1} \Delta V_i$ have the following family of distribution

$$f_{\overline{\Delta V}_i}(v) = \sum_{n=1}^{\infty} \frac{\left(\frac{2^{p-i+1} k_m v}{\alpha} \right)^{n-1}}{(n-1)!} \frac{2^{p-i+1} k_m}{\alpha} \exp\left(-\frac{2^{p-i+1} k_m v}{\alpha} \right) f_N(n), \quad i = 1, 2, \ldots, p. \tag{S4.4}$$

Note that the random variables $\overline{\Delta V}_i$ are independent but not identical anymore. However, they still follow conditional Erlang distributions same as ΔV_i and therefore inherit the scale invariance property. The distribution of sum of above Erlang random variables can be written in the following compact form [15, 16]

$$f_{V_p}(v) = \sum_{n=1}^{\infty} C_{n,p} \sum_{i=1}^{p} Q_i(v) e^{-2^{p-i+1} \frac{k_m}{\alpha} v} f_N(n), \tag{S4.5}$$

where

$$C_{n,p} = \left(2^{p-i+1} \frac{k_m}{\alpha} \right)^n, \tag{S4.6}$$

and $Q_i(v)$, for a given $N=n$, is a polynomial in v of degree $n-1$ with following form:

$$Q_i(v)=\sum_{j=1}^{n} a_{i,j,n,p} v^{j-1}, \tag{S4.7}$$

with the coefficients $a_{i,j,n,p}$ computed as

$$a_{i,j,n,p}=\frac{(-1)^{n-j}}{(j-1)!}\times \sum_{\substack{s_1+s_2+\ldots+s_p=n-j\\ s_i=0}} \prod_{\substack{l=1\\ l\neq i}}^{p} \binom{n+s_l-1}{s_l} \frac{2^{(-l+1)n}}{\left(2^{-l+1}-2^{-i+1}\right)^{n+s_l}\left(\frac{2^p k_m}{\alpha}\right)^{s_l}}. \tag{S4.8}$$

One can note that $f_{V_p}(v)$ has polynomial terms involving the coefficients 2^{p-i+1} which are multiplied by exponential terms involving -2^{p-i+1}. Therefore the probability density will converge as p becomes large though it is difficult to find the final expression to which $f_{V_p}(v)$ converges.

Scale invariance of cell size at birth and division

The scale invariance property of $f_{V_b}(v)$ can be established using equation (S4.2) using the Laplace transform of the probability density of random variables ΔV_i [3, Supplementary Information]. Denoting the Laplace transform operator by $\mathscr{L}$, we can write

$$\mathscr{L}_{V_p}(s)=\prod_{i=1}^{p}\mathscr{L}_{\overline{\Delta V}_i}(s). \tag{S4.9}$$

Because each of $\overline{\Delta V}_i$ is scale invariant, scale invariance of $f_{V_p}(v)$ for all $p\in\{1,2,\ldots\}$ holds. Thus the scale invariance of the distribution of V_b also follows.

The cell-size at division is given by $V_b+\Delta V$. Since both V_b and ΔV are scale invariat, this results in scale invariance of distribution of cell-size at division.

S5 Distribution of division times

Recall that the distribution in equation (S2.3) assumes a given newborn cell volume V_b. We can uncondition it with respect to distribution of V_b to obtain the distribution of division times (denoted by $f_{\tau_d}(t)$)

$$f_{\tau_d}(t)=\int_0^\infty f_{FPT}(t|v)\, f_{V_b}(v)\, dv, \tag{S5.1}$$

where $f_{V_b}(v)$ is the probability distribution of cell volumes at birth.

As $f_{V_b}(v)$ has an expression given by equation (S4.5) as $p\to\infty$, we can use it to obtain expression of $f_{\tau_d}(t)$. Using this relation, the division time distribution is given by

$$f_{\tau_d}(t)$$

$$=\sum_{n=1}^{\infty}\sum_{l=1}^{\infty}\sum_{i=1}^{p}\sum_{j=1}^{l} \frac{f_N(n) f_N(l) C_{l,p} a_{i,j,l,p}\left(\frac{k_m}{\alpha}\right)^{n-1} k_m e^{\alpha t}\left(e^{\alpha t}-1\right)^{n-1}}{(n-1)!}\int_0^\infty v^{n+j-1}\exp\left(-\frac{k_m v}{\alpha}\left(e^{\alpha t}-1+2^{p-i+1}\right)\right)dv \tag{S5.2}$$

$$=\sum_{n=1}^{\infty}\sum_{l=1}^{\infty}\sum_{i=1}^{p}\sum_{j=1}^{l} \frac{f_N(n) f_N(l) C_{l,p} a_{i,j,l,p}\alpha^{j+1}(n+j-1)!}{k_m^j (n-1)!}\left(\frac{e^{\alpha t}\left(e^{\alpha t}-1\right)^{n-1}}{\left(e^{\alpha t}+2^{p-i+1}-1\right)^{n+j}}\right). \tag{S5.3}$$

From equations (S4.6) and (S4.8), one can note that the parameter α appears in $C_{l,p}$ as α^{-l}, and in $a_{i,j,l,p}$ as α^{l-j}. Therefore, we can write the above expression in

$$f_{\tau_d}(t) = \sum_{n=1}^{\infty}\sum_{l=1}^{\infty}\sum_{i=1}^{p}\sum_{j=1}^{l} \frac{f_N(n)f_N(l)C'_{l,p}a'_{i,j,l,p}\alpha(n+j-1)!}{k_m^{j+1}(n-1)!}\left(\frac{e^{\alpha t}\left(e^{\alpha t}-1\right)^{n-1}}{\left(e^{\alpha t}+2^{p-i+1}-1\right)^{n+j}}\right), \tag{S5.4}$$

where $C'_{l,p} = \alpha^l C_{l,p}$, and $a'_{i,j,l,p} = \alpha^{l-j}a_{i,j,l,p}$.

To show scale invariance of the distribution upon scaling by its mean, we compute the mean division as follows.

$$\langle \tau_d \rangle = \sum_{n=1}^{\infty}\sum_{l=1}^{\infty}\sum_{i=1}^{p}\sum_{j=1}^{l} \frac{f_N(n)f_N(l)C'_{l,p}a'_{i,j,l,p}\alpha(n+j-1)!}{k_m^{j+1}(n-1)!}\sum_{s=0}^{n-1}\binom{n-1}{s}\int_0^{\infty}\frac{te^{\alpha t}\left(-2^{p-i+1}\right)^s}{\left(e^{\alpha t}+2^{p-i+1}-1\right)^{s+1}} \tag{S5.5}$$

$$= \frac{1}{\alpha}\sum_{n=1}^{\infty}\sum_{l=1}^{\infty}\sum_{i=1}^{p}\sum_{j=1}^{l} \frac{f_N(n)f_N(l)C'_{l,p}a'_{i,j,l,p}(n+j-1)!}{k_m^{j+1}(n-1)!} \times \sum_{s=0}^{n-1}\binom{n-1}{s}\frac{1}{(j+s)^2}\,{}_2F_1(j+s,j+s;j+s+1;1-2^{i-p-1}), \tag{S5.6}$$

where ${}_2F_1$ represents the hypergeometric function. The second step above was solved using Mathematica software after changing the dummy variable of the integral via substitution $z = e^{\alpha t}+2^{p-i+1}-1$. Note that the mean division time can be written as

$$\langle \tau_d \rangle = \frac{A}{\alpha}, \tag{S5.7}$$

for a constant A which basically represents the complex expression in equation (S5.6).

The scale invariant of $f_{\tau_d}(t)$ can be shown by constructing a function $g(w)$ as

$$g(w) = A\sum_{n=1}^{\infty}\sum_{l=1}^{\infty}\sum_{i=1}^{p}\sum_{j=1}^{l} \frac{f_N(n)f_N(l)C'_{l,p}a'_{i,j,l,p}(n+j-1)!}{k_m^{j+1}(n-1)!}\sum_{s=0}^{n-1}\binom{n-1}{s}\frac{e^{Aw}\left(-2^{p-i+1}\right)^s}{\left(e^{Aw}+2^{p-i+1}-1\right)^{s+1}}. \tag{S5.8}$$

In this case, we have

$$\frac{1}{\langle \tau_d \rangle}g\left(\frac{t}{\langle \tau_d \rangle}\right) = \sum_{n=1}^{\infty}\sum_{l=1}^{\infty}\sum_{i=1}^{p}\sum_{j=1}^{l} \frac{f_N(n)f_N(l)C'_{l,p}a'_{i,j,l,p}\alpha(n+j-1)!}{k_m^{j+1}(n-1)!}\left(\frac{e^{\alpha t}\left(e^{\alpha t}-1\right)^{n-1}}{\left(e^{\alpha t}+2^{p-i+1}-1\right)^{n+j}}\right). \tag{S5.9}$$

Remark: While we showed the scale invariance property of the distribution of τ_d from above analysis, the expressions here are quite convoluted. In order to approximate the moments of division time, we go back to equation (S5.1), and use an approximate expression for $f_{V_b}(v)$. One way of approximating V_b would be to use a large finite value of p. However, that doesn't simplify the expression of $f_{\tau_d}(t)$. We therefore use $f_{V_b}(v) \approx f_{\Delta V}(v)$. Even though this is a bad approximation (recall the discussion in section S4 that only their means are same), we use it because we know that probability density of V_b would also have a Erlang like expression. Using this approximation yields

$$f_{\tau_d}(t) \approx k_m e^{\alpha t}\sum_{n=1}^{\infty}\sum_{l=1}^{\infty}\frac{f_N(n)f_N(l)\left(\frac{k_m}{\alpha}\right)^{n+l-1}\left(e^{\alpha t}-1\right)^{n-1}}{(n-1)!(l-1)!}\int_0^{\infty}v^{n+l-1}\exp\left(-\frac{k_m v}{\alpha}e^{\alpha t}\right)dv \tag{S5.10}$$

$$= \sum_{n=1}^{\infty}\sum_{l=1}^{\infty}\frac{f_N(n)f_N(l)(n+l-1)!}{(n-1)!(l-1)!}\alpha\frac{\left(e^{\alpha t}-1\right)^{n-1}}{\left(e^{\alpha t}\right)^{n+l-1}} \tag{S5.11}$$

$$= \sum_{n=1}^{\infty}\sum_{l=1}^{\infty}\frac{f_N(n)f_N(l)(n+l-1)!}{(n-1)!(l-1)!}\alpha\sum_{i=0}^{n-1}\binom{n-1}{i}(-1)^i e^{-\alpha t(l+i)}. \tag{S5.12}$$

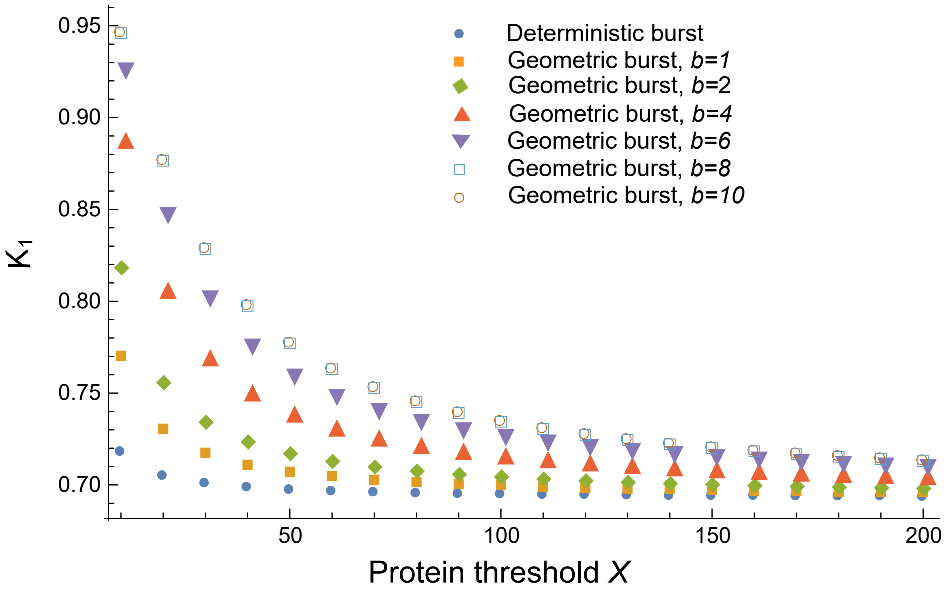

Figure S2: Values of the constant K_1 for different values of protein threshold X, and mean burst sizes b.

Moments of division time τ_d can be written as

$$\left\langle \tau_d^j \right\rangle \approx \frac{j!}{\alpha^j} \sum_{n=1}^{\infty} \sum_{l=1}^{\infty} \frac{f_N(n) f_N(l)(n+l-1)!}{(n-1)!(l-1)!} \sum_{i=0}^{n-1} \binom{n-1}{i} \frac{(-1)^i}{(i+l)^{j+1}} \tag{S5.13}$$

$$= \frac{K_j}{\alpha^j}, \tag{S5.14}$$

where constants K_j are shorthand for the summation terms.

As expected from a deterministic analysis, and approximate computations performed in [3], the constant K_1 should be approximately equal to $\log 2$. We found that $K_1 \approx 0.7 \approx \log 2$ for the two distributions of burst size B_i (deterministic, and geometric) used in this manuscript, and several values of threshold X. Some of these values are shown in Figure S2.

S6 Moments of cell-division time given newborn cell size

In this section, we provide details on how FPT (cell division time) moments depend on cell size at birth V_b. The result pertaining dependence of mean FPT on V_b is provided here. How change in V_b affects the noise in FPT has been described in the main text (Figure 2).

Mean cell-division time as a function of newborn cell size

The expression for FPT probability density in equation (S2.3) can be used to numerically compute the mean FPT as V_b is varied. The model predicts that the mean division time decreases as the newborn cell volume is increased. This behavior is consistent with previous understanding of negative correlation between cell division time and newborn cell size [2, 3].

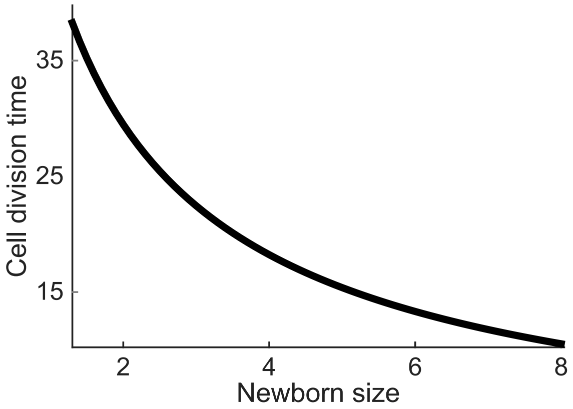

Figure S3: *The division time decreases as the cell size at birth increases*. The plot shows the division time (mean FPT) given a newborn volume computed numerically for each value of initial cell volume V_b. The protein production is assumed to be in geometric bursts and the model parameters used are $k_m = 0.13\ \text{min}^{-1}$, $X = 65$ molecules, $\alpha = 0.03\ \text{min}^{-1}$, and $b = 5$ molecules.

Inference of model parameters

Here we discuss how model parameters, namely, transcription rate (k_m), the threshold (X), and the protein burst size (b) used to draw Figure 2a in the main text were inferred. We used the moments of the added volume ΔV to estimated the model parameters from experimental data. The reason behind this is that exact expressions of the moments of added volume are available (equations (S3.10)–(S3.12)). To compute the estimates, we use the nonlinear constrained optimization solver (fmincon) available in MatLab ® with 0.01 as initial parameter value for k_m, X, and b. The estimated values for the transcription rate k_m, protein threshold X, and mean burst size b were 0.13, 65.0, and 5.0, respectively.

Data processing to obtain Fig. 2b

Since the initial size of the cell is a real random variable, we used a binning strategy to get cell cycle time statistics (noise) given different newborn sizes. We organized the newborn cell sizes into four bins: $1-2.8\ \mu m$, $2.8-4.5\ \mu m$, $4.5-6.3\ \mu m$, and $6.3-8\ \mu m$. For each bin, we computed confidence intervals of the noise in cell cycle time using bootstrapping. The results show significant increments in the noise of cell cycle time (no overlapping confidence intervals) as the mean initial size in the bin increases. This behavior is also seen if the number of bins is varied from four to six, or eight. However, we only show the four-bins case in the manuscript because larger number of bins introduces larger errors bars (confidence interval width four times larger that the median cell cycle time noise) in the smallest/largest bins because of small number of cells in them.

References

[1] W. J. Voorn and L. J. H. Koppes. Skew or third moment of bacterial generation times. *Archives of Microbiology*, 169:43–51, 1997.

[2] Manuel Campos, Ivan V. Surovtsev, Setsu Kato, Ahmad Paintdakhi, Bruno Beltran, Sarah E. Ebmeier, and Christine Jacobs-Wagner. A constant size extension drives bacterial cell size homeostasis. *Cell*, 159:1433–1446, 2014.

[3] Sattar Taheri-Araghi, Serena Bradde, John T. Sauls, Norbert S. Hill, Petra Anne Levin, Johan Paulsson, Massimo Vergassola, and Suckjoon Jun. Cell-size control and homeostasis in bacteria. *Current Biology*, 25:385–391, 2015.

[4] João Pedro Hespanha and Abhyudai Singh. Stochastic models for chemically reacting systems using polynomial stochastic hybrid systems. *International Journal of Robust and Nonlinear Control*, 15:669–689, 2005.

[5] Laurence A. Baxter. Reliability applications of the relevation transform. *Naval Research Logistics*, 29:323–330, 1982.

[6] Franco Pellerey, Moshe Shaked, and Joel Zinn. Nonhomogeneous poisson processes and logconcavity. *Probability in the Engineering and Informational Sciences*, 14:353–373, 2000.

[7] Otto G Berg. A model for the statistical fluctuations of protein numbers in a microbial population. *Journal of Theoretical Biology*, 71:587–603, 1978.

[8] David R. Rigney. Stochastic model of constitutive protein levels in growing and dividing bacterial cells. *Journal of Theoretical Biology*, 76:453 – 480, 1979.

[9] Nir Friedman, Long Cai, and X Sunney Xie. Linking stochastic dynamics to population distribution: an analytical framework of gene expression. *Physical Review Letters*, 97:168302, 2006.

[10] Abhyudai Singh, Brandon Razooky, Chris D Cox, Michael L Simpson, and Leor S Weinberger. Transcriptional bursting from the HIV-1 promoter is a significant source of stochastic noise in HIV-1 gene expression. *Biophysical Journal*, 98:L32–L34, 2010.

[11] Abhyudai Singh and Pavol Bokes. Consequences of mRNA transport on stochastic variability in protein levels. *Biophysical Journal*, 103:1087–1096, 2012.

[12] Abhyudai Singh, Brandon S Razooky, Roy D Dar, and Leor S Weinberger. Dynamics of protein noise can distinguish between alternate sources of gene-expression variability. *Molecular Systems Biology*, 8:607, 2012.

[13] Khem Raj Ghusinga and Abhyudai Singh. First-passage time calculations for a gene expression model. In *IEEE 53rd Annual Conference on Decision and Control (CDC)*, pages 3047–3052, 2014.

[14] Abhyudai Singh and John J Dennehy. Stochastic holin expression can account for lysis time variation in the bacteriophage λ. *Journal of The Royal Society Interface*, 11:20140140, 2014.

[15] Carlos A Coelho. The generalized integer Gamma distribution–a basis for distributions in multivariate statistics. *Journal of Multivariate Analysis*, 64:86–102, 1998.

[16] H Jasiulewicz and W Kordecki. Convolutions of Erlang and of Pascal distributions with applications to reliability. *Demonstratio Mathematica*, 36:231–238, 2003.

[17] Ping Wang, Lydia Robert, James Pelletier, Wei Lien Dang, Francois Taddei, Andrew Wright, and Suckjoon Jun. Robust growth of Escherichia coli. *Current Biology*, 20:1099–1103, 2010.
